# Is Dentistry a Specialty of Medicine?

**Published:** 1887-01

**Authors:** W. A. Purrington


### Is Dentistry a Specialty of Medicine?

"BARBARA, CELARENT, DARII, FERIOQUE PRIORIS."

TO THE EDITOR OF THE MEDICAL RECORD.

*Sir:*—Inextinguishable laughter must have shaken the sides of the less reverential disciples of the Academy when the mad wag of Sinope threw the plucked cock among them and roared, "Behold Plato's man!" And perhaps it was this sardonic criticism of his definition of man as a featherless biped that caused the master to say—if he said it—“ Show me one who can define, and I will show you a god.”

We cannot say too often that knowledge is precise in proportion to the accuracy of the definitions upon which it rests. A precise knowledge of what the term “judicial opinion” means, for instance, would have prevented the periodical *Science* from making a grave misstatement of fact in its issue of November 19th, which contained a disparaging editorial note, apropos of a recent prosecution by the Medical Society of the County of New York, which surely would not have appeared if the writer had been careful in testing the accuracy of his facts and definitions. And it is just because of the difficulty of clearly defining terms, and accurately laying down premises, that the law is so uncertain; almost as uncertain as other inexact sciences.

A stray sentence in a recent article of mine on medical legislation has enticed into the perilous mazes of definition the President of the New York State Dental Society, Norman W. Kingsley, D.D.S., who maintains that a doctor of dental surgery, if that be the connotation of the mystic letters following his name, is so called because he is neither a doctor of medicine nor a surgeon, neither a plumber nor a sculptor, but is a dentist—that is to say, a betwixt-and-between, compound of all these simples.

It is only because every discussion may be of use that tends to clearly define, in a legal sense, what constitutes the practice of medicine, and not from any joy of conflict, or sympathy with the late pestilent Mr. Michael Brady’s wife, who argued because it made conversation, that I venture to consider whether the President’s “ therefore,” in the first line of paragraph five, in his letter to THE RECORD of November 20th, is a *sequitur*, and whether there is wrapped up in it a syllogism reducible to any of the forms rendered dear to logical minds by the simple quatrain beginning:

> “ Barbara, Celarent, Darii, Ferioque prioris,
> Cesare Camestres Festino Baroko, secundæ.”

I must strenuously repudiate any purpose of belittling dentistry as a calling; in fact, by holding it to be, in so far as it is a profession, a branch of medicine, I extol it. And if Plato failed to define a man, it cannot be considered a reflection on President Kingsley to point out that he has failed to define a dentist, who is, as I presume will be admitted, a species of man, and not a distinct creation.

Before we arrive at this pregnant "therefore," a word that indicates the bringing to bed of a logical argument, which, after proper gestation, is about to be delivered of a conclusion, the premises must be examined, and a diagnosis made, lest the swelling word prove turgid from other causes, and the wise women smile at us, recalling the verse of Omar:

> "We came like water, and like wind we go."

The premises of the President are to be found in the first four paragraphs of his letter, and, briefly stated, appear to be as follows:

First. Dentistry covers every department known under that name, and he alone is truly a dentist who can practise every specialty of it.

Second. A dentist may be an oral surgeon, or several other things, *e. g.*, an anatomist, a physiologist, a mechanic, "but no one of these practised to perfection makes him a dentist." *

Third. Oral surgery is a specialty of dentistry.

Fourth. Oral surgery is not dentistry.

Such is intended to be, and I think is, a fair statement of the contents of the four premise-containing paragraphs; and, after reading them and the locally sequent, "I *therefore* affirm that dentistry is not a specialty of any other science or art, but is a profession in itself," one is fully justified in saying "Great Scot!" †

---

* Here a fallacy; our disreputable old friend the *petitio principii* lifts his head, which we must deal a lusty blow right off. Our contention is that a perfect tooth-mechanic is an ideal dentist in every sense in whieh the dentist is not a practitioner in a limited field of medicine.

† The esoteric sense of the initiates will perceive at once that this is not slang, as the vulgar might suppose. Reference is made to John Scotus Erigena; and the shade of that greatest of schoolmen and dialectors is invoked, most reverentially, to illumine an age that, by neglect of his favorite studies, has come to consider asseveration as the same thing with demonstration.

Suppose one of the worthy class of citizens who, with some justice, call themselves tonsorial artists, but whose entitlement of their art is a profession the President declares to be an assumption, the class, to wit, of the barbers, from the efficient loins of whose mystery sprang the surgeons—suppose, I say, that the Chief Master of their guild should argue thus:

First. Barberism * covers every department known under that name, and he alone is a barber in very truth who is able not only to depilate, shear, and adorn, but also by removing scurf, dandruff, and all morbific conditions of the scalp; to pilate, if we may so say, the human head,† which is greater than the teeth, as containing them.

Second. A barber may be a mechanic, *e. g.*, a maker of switches, Thompson waves, and other hirsute deceptions of the lovelier sex; he may be a dermatologist, a clever chemical compounder of hairine, bandoline, and other articles terminating in "ine;" a proprietor of baths; above all, a brilliant conversationalist, but none of these callings, not even the last, though practised in perfection, makes him a barber, *teres atque rotundus.*

Third. Shaving is a specialty of barberism.

Fourth. Barberism is not shaving.

Ergo. I affirm that barberism is not a specialty of any other science or art, but is a profession in itself.

Would the President call this argument?

"But "Dentistry," says the President, "and Barberism too," might add the Chief Master, "is a profession, because it is a vocation of beneficence." Well, and what is plumbing, by similar reasoning?

But, to be more serious: let it be admitted that between the President and myself there is no ground on which to differ unless we are to argue for conversation's sake. Zeal for the cause and haste of composition have led him into a chain of statements that

---

* Alas that the language supplies no worthier name!

† A young lady of Chicago once amused me by further enlarging the modern barber's function and restoring his lost surgical powers. Returning from having her tonsils cut, she said, in reply to a question as to her whereabouts, "I have just been having a tonsorial operation performed."

will not bear analysis as argument, but constitutes none the less about as good an array of words as can be mustered to defend so weak a thesis as his, viz., that the part is distinct from and as great as the whole. His excellent sense wipes away the sophistry of his sympathies and admits absolutely my contention; for he says: "That which dignifies the practice of dentistry, bringing it above ordinary mechanics, is the fact that the operations are performed upon living organisms, *and that which makes it professional* is the *knowledge of anatomy, pathology, etc., which discriminates in directing the mechanical treatment.*" There is the whole matter in a nutshell. Just so far as dentistry is not a purely mechanical handicraft, it is a branch of medicine. So long as the surgeon was only a blacksmith with a searing-iron, so long as the barber-surgeon mechanically applied a bandage, a cup, or a leech, and nothing more, they were tradesmen, that is to say, "mechanics or artificers whose livelihood depends upon the labor of their hands," and not professional men, that is to say, men deriving their livelihood from the application of abstract knowledge and reasoning thereon to the concrete affairs of life.

The fact that its operations are performed on living organisms does not, *per se*, dignify the dentist's calling over that of the chiropodist, manicure, masseur, or barber, any more than the fact that its operations are performed on inorganic matter belittles the profession of a civil engineer. Nor does the fact that the extraction, filling, and imitation of teeth are carried on chiefly as mechanical operations affect the truth of my statement, excepted to by President Kingsley, that "when the dentists, ceasing to be mechanics, undertake the treatment of diseases of the mouth, they become practitioners of medicine and surgery."

President Kingsley, I assume, would not accept the reasoning of that Caucasian variety of the "Kickapoo Indian," who tried to convince the court recently that a registered dentist had a right to prescribe for a patient's liver, or other diseased organ, if he considered the teeth affected by it. And on my part I admit that the dentist's calling is so largely mechanical in its processes that, just as was that of the surgeon in times past, it has been denied by some to form part of the medical profession. Thus, Webster defines a dentist as "one who makes it his business to clean, extract, and repair natural teeth, and to insert artificial ones." This would make the dentist an artisan. Dunglison, however, in his "Dictionary," * recognized by his definition the calling of dentistry as entirely professional, for he defined a dentist as "one who devotes himself to the study of the diseases of the teeth and their treatment." Neither definition is accurate. The courts also have tried to throw some light on the question of how a dentist should be rated. I will cite three cases only. In Lee *vs.* Griffin (1 E. B. and S., 272) it appeared that a lady, after having ordered two sets of teeth, for the better making of which a model of her mouth had been made, died before she tried them in, but not until after a day had been appointed for her to inspect them. The executors, not considering the teeth as valuable assets, declined to pay for them. The dentist sued them for 21 ℔., and his counsel urged that what the deceased contracted for was the dentist's skill, and that the materials on which it was exerted were but incidents to the employment—the case being analagous to that of an artist employed to make a picture. But the court said: "The subject matter of the contract was the supply of goods. The case bears a strong resemblance to that of a tailor supplying a coat, the measurement of the mouth and the fitting of the teeth being analagous to the measurement and fitting of the garment." So the plaintiff lost his case in spite of his teeth.

In Michigan the taxation of a dentist's instruments as mechanics' tools was upheld, and the court said that "a dentist, in one sense, is a professional man, in another sense his calling is mainly mechanical, and the tools which he employs are used in mechanical operations." † In Mississippi a contrary view was taken, the court saying: "A dentist cannot be properly denominated a mechanic. It is true that the practice of his art requires the use of instruments, . . . but it also involves a knowledge of the physiology of the teeth, which cannot be acquired but by a proper course of study, and this is taught by learned treatises on the subject, and as a *distinct though limited part of the medical*

* Edition of 1866.

† Maxon *vs.* Perrott, 17 Mich., 332.

*art.* . . . If such persons should be included in the denomination of mechanics, because their pursuit required the use of mechanical instruments, . . . the same reason would include general surgeons under the same denomination. . . ." *

The function of dentistry being as yet chiefly mechanical, and carried on in well-defined limits, in all the bills recently brought forward by the Medical Society of the State, dentists have been expressly exempted from purview; and it will be the questions arising in civil cases, chiefly those for damages resulting from bad work and advice, and not criminal actions, that will eventually settle in what degree the dentist is to be regarded as a professional man. Such a question might arise thus: A, a pretty girl, sues B, a dentist, for damages resulting from an operation whereby her beauty and, incidentlly, her lover have been lost. This is an actual, not an imaginary case, and on its threshold the question arises how far did B act as a practitioner in a limited field of medicine, and how far, as a mechanic, is he exempt from liability for ignorance of results that medical education would have taught him might flow from his manual operation.

I am, Sir, your obedient servant,

W. A. PURRINGTON.

